# Evolutionary trajectory of SARS-CoV-2 and emerging variants

**DOI:** 10.1186/s12985-021-01633-w

**Published:** 2021-08-13

**Authors:** Jalen Singh, Pranav Pandit, Andrew G. McArthur, Arinjay Banerjee, Karen Mossman

**Affiliations:** 1 School of Interdisciplinary Science, McMaster University, Hamilton, ON Canada; 2 EpiCenter for Disease Dynamics, One Health Institute, School of Veterinary Medicine, University of California Davis, Davis, CA USA; 3 Department of Biochemistry and Biomedical Sciences, McMaster University, Hamilton, ON Canada; 4 Michael G. DeGroote Institute for Infectious Disease Research, McMaster University, Hamilton, ON Canada; 5 Vaccine and Infectious Disease Organization, University of Saskatchewan, Saskatoon, SK Canada; 6 Department of Veterinary Microbiology, Western College of Veterinary Medicine, University of Saskatchewan, Saskatoon, SK Canada; 7 Department of Biology, University of Waterloo, Waterloo, ON Canada; 8 Department of Medicine, McMaster University, Hamilton, ON Canada; 9 McMaster Immunology Research Centre, McMaster University, Hamilton, ON Canada

**Keywords:** SARS-CoV-2, Coronavirus, Evolution, Mutations, Selection, Variants

## Abstract

The emergence of a novel coronavirus, severe acute respiratory syndrome coronavirus 2 (SARS-CoV-2), and more recently, the independent evolution of multiple SARS-CoV-2 variants has generated renewed interest in virus evolution and cross-species transmission. While all known human coronaviruses (HCoVs) are speculated to have originated in animals, very little is known about their evolutionary history and factors that enable some CoVs to co-exist with humans as low pathogenic and endemic infections (HCoV-229E, HCoV-NL63, HCoV-OC43, HCoV-HKU1), while others, such as SARS-CoV, MERS-CoV and SARS-CoV-2 have evolved to cause severe disease. In this review, we highlight the origins of all known HCoVs and map positively selected for mutations within HCoV proteins to discuss the evolutionary trajectory of SARS-CoV-2. Furthermore, we discuss emerging mutations within SARS-CoV-2 and variants of concern (VOC), along with highlighting the demonstrated or speculated impact of these mutations on virus transmission, pathogenicity, and neutralization by natural or vaccine-mediated immunity.

## Background

Coronaviruses (CoVs) can infect humans and animals to cause mild to severe disease, including death [1]. CoVs are divided into four genera: *alpha-* and *beta-CoVs* predominantly originate in bats and infect other mammals, while *gamma-* and *delta-CoVs* originate in and largely infect avian species [2]. CoV infection in animals is generally associated with gastric symptoms [3], such as acute diarrhea in young pigs that are infected with porcine epidemic diarrhea virus (PEDV) and swine acute diarrhea syndrome coronavirus (SADS-CoV) [4, 5]. While CoVs mainly circulate in animals, such as pigs, camels, cats, and bats [6], there have been at least seven documented instances where these viruses have spilled over into humans [7]. These events have led to the emergence of human coronaviruses (HCoVs) that are low and high pathogenic. The origin of the most recently emerged human coronavirus, severe acute respiratory syndrome coronavirus 2 (SARS-CoV-2) is speculated to be associated with Rhinolophus bats, but the zoonotic transmission pathway remains unknown.

HCoV-229E, HCoV-OC43, HCoV-NL63 and HCoV-HKU1 represent endemic and low pathogenic HCoVs, and are responsible for one-third of common cold symptoms [8]. High pathogenic HCoVs such as severe acute respiratory syndrome coronavirus (SARS-CoV), Middle East respiratory syndrome coronavirus (MERS-CoV), and SARS-CoV-2 cause or have caused severe disease in humans with case-fatality rates of 10.9%, 34.3%, and 2.1%, respectively [9–11]. SARS-CoV, MERS-CoV and SARS-CoV-2 are *beta-CoVs* [12, 13]. MERS-CoV belongs to the *Merbecovirus* subgenus, while SARS-CoV and SARS-CoV-2 belong to the SARS-related coronavirus (SARSr-CoV) species within the *Sarbecovirus* subgenus [14]. It remains unclear why most HCoVs evolved to largely cause minor illness while MERS-CoV continues to cause severe disease [15–17]. In this review, we have highlighted the origins of HCoVs and mapped positively selected for mutations within HCoV proteins to discuss the evolutionary trajectory of SARS-CoV-2. We have also discussed emerging mutations within SARS-CoV-2 and variants of concern (VOC), along with highlighting the demonstrated or speculated impact of these mutations on virus transmission, pathogenicity, and neutralization by natural or vaccine-mediated immunity.

## Origin of human coronaviruses

**Fig. 1:**
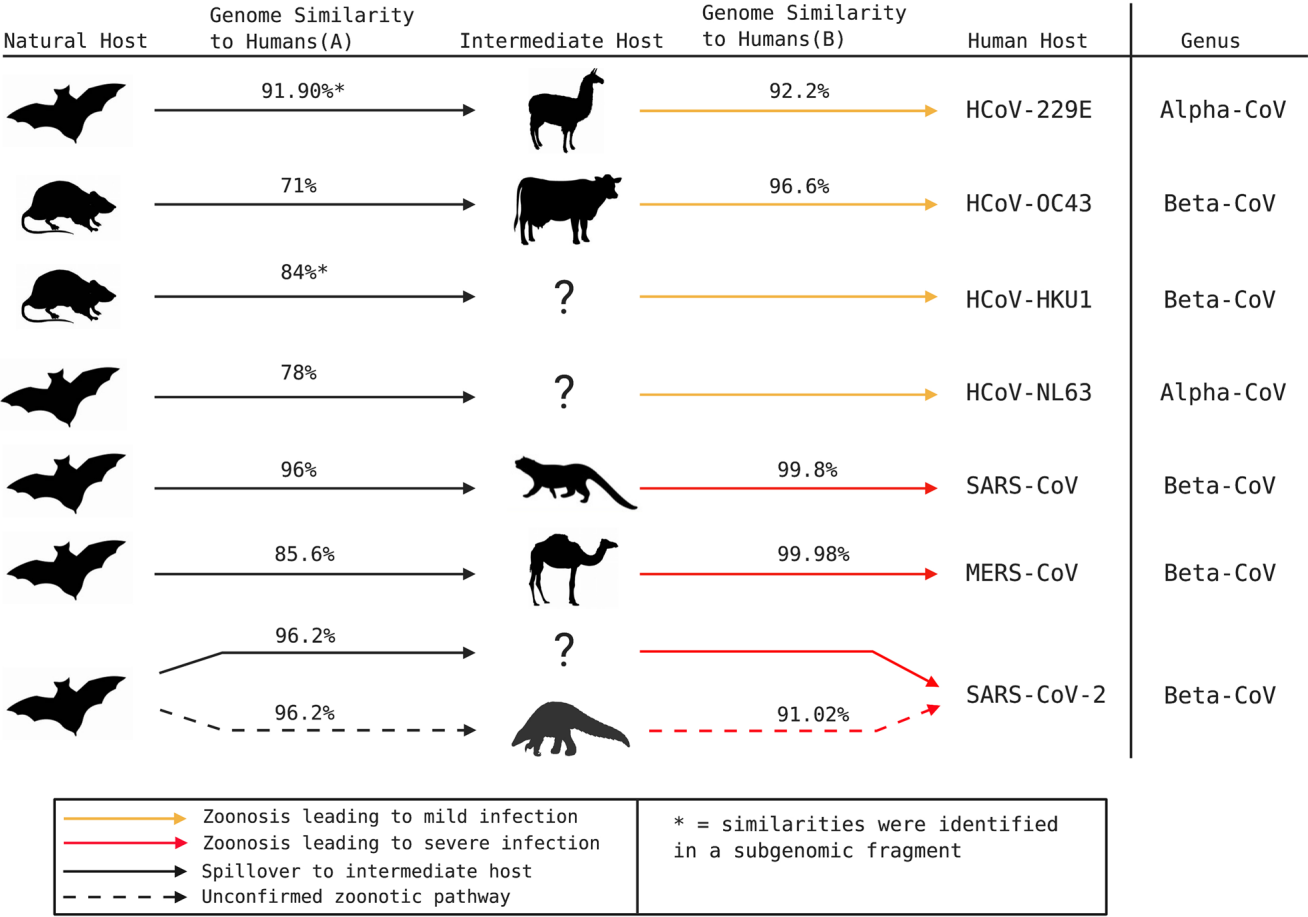
Speculated animal origins of known human coronaviruses. HCoV species are organized chronologically (top to bottom) by their speculated dates of spill over into humans. Intermediate hosts (top to bottom) shown are alpacas, cattle, civet cats, dromedary camels, pangolins, and unknown (denoted as a question mark). Genome similarity to humans (A) indicates percentage similarity of CoV genomes detected in reservoir species with corresponding human CoV. Genome similarity to humans (B) indicates percentage similarity of CoV genomes detected in intermediate species with corresponding human CoV. Non-human CoVs that are highly pathogenic in animals, such as PEDV and SADS-CoV, are not shown here. Genomic percentage similarities were extracted from existing primary studies [20, 21, 32, 56, 60, 277–283]

All known HCoVs are speculated to have an evolutionary origin in bats or rodents [1, 3, 18] (Fig. 1), with five of seven HCoVs originating in bats [3, 19–21] (Table 1). Bats are speculated to be primordial hosts for all CoV lineages due to ubiquitous detection of diverse CoVs and constant CoV population growth, which contrasts epidemic-like growths observed in other animals [22]. Although bats and alpacas can serve as MERS-CoV reservoirs [23, 24], dromedary camels are the major reservoir host and primary contributor to human infections [25–28] (Fig. 1). The full extent of wildlife or intermediate animal reservoirs of SARS-CoV-2 is currently unknown.

**Table 1:** Speculated timelines for evolutionary origins of known human coronaviruses from bats

Species	Discovery in humans	Speculated timeline of divergence for human strain	Speculated bat reservoir	References
SARS-CoV-2	2019	Human strain likely diverged from most closely related bat virus in 1969	*Rhinolophus* spp.	[32, 294, 295]
SARS-CoV	2003	Human strain likely diverged from bat strain in 1986	*Rhinolophus* spp.	[22, 53, 280, 296–298]
MERS-CoV	2012	Human strain likely diverged from bat strain before 1990	*Taphozous perforates, Pipistrellus* spp., *Neoromecia* spp.	[282, 299–305]
HCoV-OC43	1967	Human strain likely diverged from bovine strain in 1890	N/A	[276, 306]
HCoV-HKU1	2004	No supported dates of divergence have been established	N/A	[279]
HCoV-229E	1965	Human strain likely diverged from alpaca strain before 1960 and from bat strain between 1686 and 1800 CE	*Hipposideros caffer ruber*	[21, 56, 307]
HCoV-NL63	2004	Human strain likely diverged from bat strain between 1190 and 1449 CE	*Triaenops afer*	[20, 308–310]

SARS-CoV-2 is believed to have originated in a seafood market in Wuhan, Hubei Province, China [29], although limited contact-tracing at the beginning of the pandemic does not allow for definitive characterization of the exact events that led to the first human-to-human transmission, including the index patient or initial animal contact. Nonetheless, it is speculated that the natural reservoirs of SARS-CoV-2 are *Rhinolophus* bats (Table 1) since diverse SARSr-CoVs have been detected in multiple *Rhinolophus* species [22, 30, 31], including RaTG13 in *R. affinis* [32]. RaTG13 is 96.2% identical to SARS-CoV-2 at the whole genome level [32]. Moreover, SARS-CoV-2 contains a polybasic furin-like cleavage site between S1 and S2 spike (S) protein subunits, similar to *Rhinolophus* CoV RmYN02 [33, 34], which shares 93.3% whole genome nucleotide identity with SARS-CoV-2 [34]. However, the receptor binding domain (RBD) of SARS-CoV-2 is only 85% and 61.3% identical to those of RaTG13 and RmYN02, respectively [34–36]. RaTG13 and RmYN02 were discovered in bats of China’s Yunnan province, over 1500 km away from Wuhan [34, 35]; however, this does not preclude the possibility of virus spill over as bats can fly long distances. Virus transmission and transport by susceptible intermediate reservoirs or humans is also possible.

Phylogenetic analyses have identified a possible recombination-mediated origin for SARS-CoV-2 [37–39]. Neutralizing antibodies to SARS-CoV and SARS-CoV-2 have been detected in Malayan pangolins (*Manis javanica*), suggesting that SARSr-CoVs have been circulating in pangolins since 2003 [40]. Recombination of CoVs within Malayan pangolins has been suggested given the 97.4% amino acid similarity within the RBDs of pangolin SARSr-CoVs and SARS-CoV-2 [35, 41], including conservation of all critical residues required for successful human ACE2 (hACE2)-mediated cellular entry [35, 39, 41, 42] and the detection of pangolin SARSr-CoVs that bind to hACE2 [43]. Additionally, bats and pangolins may share underground caves [44], facilitating ecological contact in high density areas. However, the lack of robust evidence of direct SARS-CoV-2 emergence from a pangolin CoV precursor [45], along with the reported high pathogenicity of SARSr-CoVs in infected pangolins [41, 42, 45] makes it unlikely that pangolins are intermediate reservoirs of SARSr-CoVs.

The nucleotide percentage similarity of CoVs detected in reservoir species is generally lower than CoVs detected in intermediate species. Adaptive evolution of CoVs in intermediate species facilitates successful spill over into humans (Fig. 1). Since SARS-CoV-2 is more closely related to bat SARSr-CoVs than to pangolin SARSr-CoVs (Fig. 1), it seems unlikely that pangolins are intermediate hosts, unless we haven’t yet detected the full range of SARSr-CoVs in pangolins. It is uncertain whether an unknown intermediate host provided an opportunistic amplifying role or a stable reservoir for the zoonotic transmission of SARS-CoV-2.

While direct human infection with bat SARSr-CoVs has not been reported [46], it is possible that the majority of adaptive evolution of SARSr-CoVs occurs in bats, prior to spill over into humans [47]. Some notable adaptations include carrying the lowest level of CpG dinucleotides among known *beta-CoV* genomes [48], similar to a mechanism of escaping innate immunity observed in camel MERS-related CoVs strains [49, 50]. The relatively few SARSr-CoVs detected in the Hubei Province [35] are phylogenetically distant from SARS-CoV-2 [51]. Indeed, if SARS-CoV-2 did transmit from animals to humans, further sampling in Hubei Province may identify more closely related SARSr-CoVs in archived animal specimens. Investigating the possibility of an infected person travelling to Wuhan and unwittingly spreading the virus will be more difficult in the absence of archived samples and records of travel history.

Despite the abundance of SARSr-CoVs and *beta-CoVs* in bat species [52, 53], it is likely that additional reservoirs and intermediate hosts remain undetected [54]. Pigs, alpacas, and dromedary camels also maintain a variety of CoVs with the potential to transmit to humans [3, 12, 20, 55–57]. Independent insertions within RBDs of SARS-CoV, MERS-CoV, and SARS-CoV-2 suggest convergent evolution, which will likely lead to emergence of more pathogenic HCoVs [58]. Further sampling of bats, pangolins, and other species that share an ecological niche with bats may help piece together the puzzle surrounding the spill over of SARS-CoV-2 into humans [59] and also help discover other CoVs with potential to infect humans.

Aside from consistent spill over of MERS-CoV from camels [60], HCoVs have emerged through limited spill over events, followed by human-to-human transmission [3, 61]. While challenging to predict, future spill over events are likely, due to the long history of CoV host shifting [62–65]. Anthropogenic factors such as urbanization and deforestation increase habitat overlap of humans and animals, providing increased zoonotic transmission opportunities [57, 66]. Areas of high contact between humans, wildlife, and domesticated animals, such as live animal wet markets provide opportunity for viral recombination and adaptation to a broader range of animal species prior to transmission to humans [57]. Identifying existing CoV diversity in such areas will enhance our understanding of ecological opportunities for zoonosis and will help us better predict and prevent the emergence of future HCoVs.

## Evolution of SARS-CoV-2 and its variants

**Fig. 2:**
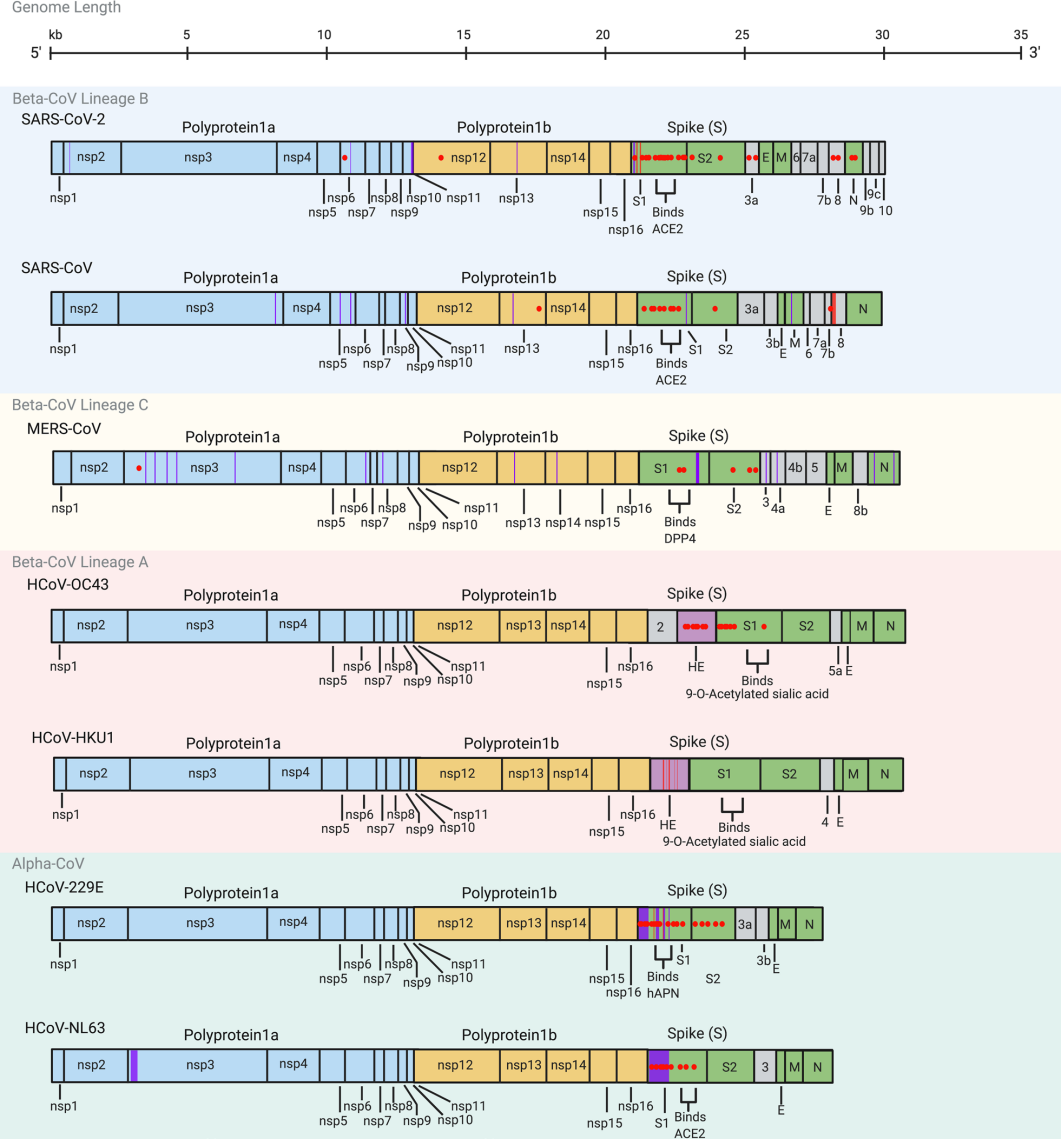
Mutations identified in human coronaviruses. Red dots within the genomes correspond to specific amino acid residues that have been strongly positively selected for such that a specific mutation has become dominant in the region where it emerged [74, 78, 83–91, 94–96, 99–101, 104, 111, 116, 117, 121, 123–125, 129, 131, 132, 135, 138–140, 146, 151–154, 158, 162, 278, 284–293]. Genomic regions highlighted by red bars correspond to deletions that have been selected for, while purple bars correspond to regions with significant polymorphisms within a CoV species. *Beta-CoV* Lineage B (*Sarbecovirus*) is represented within the blue shaded area, *beta-CoV* Lineage C (*Merbecovirus*) is represented within the yellow shaded area, *beta-CoV* Lineage A (*Embecovirus*) is represented within the red shaded area, and *alpha-CoVs* are represented within the green shaded area. Genome length in kilobases (kb) is noted on top. See Table 2 for more details

Co-evolution of CoVs with their hosts is driven by genetic diversity that is selected through evolutionary pressures. CoV genetic diversity is made possible by a large genome (26.4–31.7 kb) [67], high mutation rate due to a low fidelity viral polymerase (~ 10^–4^ substitutions per site per year) [68, 69], and high recombination frequency (up to 25% for the entire genome in vivo) [70, 71]. Mutations that confer greater fitness are selected for, leading to antigenic drift. Ratios of the rates of non-synonymous/synonymous mutations (*dN/dS*) greater than one, less than one and equal to one indicate positive selection, negative (purifying) selection and neutral evolution, respectively [72]. SARS-CoV-2 genomes are currently under purifying selection [73, 74]. Despite observing little viral diversity at the beginning of the COVID-19 pandemic [75, 76], positive selection with presumed advantages such as increased transmission rates has now been documented [77–79] (Fig. 2, Table 2). However, functional characterization of these mutations remains under-investigated.

**Table 2:**
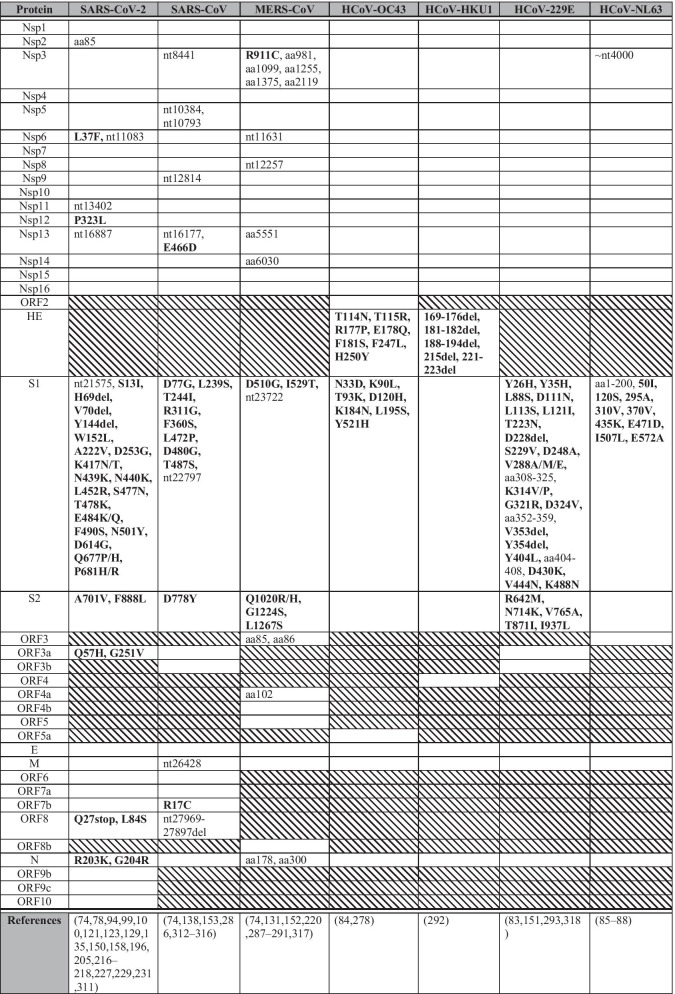
Selection sites across various human coronaviruses

This table illustrates positively selected for residues across multiple human coronaviruses. Shaded boxes represent proteins not encoded by the specific CoV species. Text in bold highlight mutations and deletions that were positively selected for and showed population-level expansion, while non-bolded text represents highly polymorphic sites. Sites are indicated as nucleotide (nt) position or amino acid (aa) position. Empty cells in the table represent lack of evidence for positive selection or lack of publications on positive selection within these regions

Antigenic drift is most frequently observed in viral surface proteins that are highly exposed to selection pressures of the immune system, such as neutralizing antibodies [80]. Indeed, CoV spike genes, particularly the S1 and RBD coding regions, have the highest detected non-synonymous mutation rates [81, 82], a trend observed across the majority of HCoVs (Fig. 2). For low pathogenic and endemic HCoVs, multiple positively selected for residues and polymorphic sites are found in the N-terminal domain (NTD) of S [83–88]. A notable exception is HCoV-HKU1, for which there is a shortage of sequencing data outside of the hemagglutinin esterase (HE) gene. Emerging data suggest that positively selected for and homoplastic sites have been observed within the SARS-CoV-2 NTD as well [78, 89–91]. Given the observations with other HCoVs (Fig. 2) and the detection of neutralizing epitopes within the SARS-CoV-2 NTD [91, 92], we speculate that with continued circulation, vaccination and convalescent sera therapy, further positively selected for mutations in the NTD are likely to occur. Further retrospective research on the evolution of endemic HCoVs may help predict the likely evolutionary trajectory of SARS-CoV-2.

CoV genomic mutations give rise to virus variants, and closely related variants are grouped into clades. SARS-CoV-2 variants have been clustered into nine clades: L, V, S, G, GH, GR, GV, GRY and O [93, 94] (Table 3), named after their most representative mutations [95]. Clade L dominated the beginning of the pandemic [38], prior to the appearances of clade S and the less defined clade O in early January, 2020 [73, 93, 96]. Clades V and G appeared in mid-January, followed by clades GH and GR at the end of February, clade GV at the end of June, and clade GRY in September, 2020 [94, 97, 98]. Clades L and V are likely extinct, while clades G, GH, GR, and GRY comprise the majority of global SARS-CoV-2 sequences currently [97, 98]. Clade S has also been declining since the emergence of clade G [93]. Following rapid dissemination of clade G and its derivatives, such as B.1.1.7, B.1.351, P.1, and B.1.617.2 variants (Table 5), we may see the rise of other variants, selected by mounting population-level immunity and other yet unidentified factors [89, 99–101], highlighting the need for international genome surveillance efforts and global data sharing via the established GISAID resource [102].

**Table 3:** Characteristic mutations detected in circulating SARS-CoV-2 clades

Clade	Characteristic mutations	References
L	Reference Genome NC_045512.2	[94, 319]
V	Nsp6: L37F ORF3a: G251V	[95, 123, 129, 284]
S	Nsp4: S76S ORF8: L84S	[96, 285]
G	5’ UTR: C241T * Nsp3: F106F Nsp12: P323L S: D614G	[104, 116, 117]
GH	5’ UTR: C241T * Nsp3: F106F Nsp12: P323L S: D614G ORF3a: Q57H	[121, 146]
GR	5’ UTR: C241T * Nsp3: F106F Nsp12: P323L S: D614G N: R203K N: G204R	[123]
GV	5’ UTR: C241T * Nsp3: F106F Nsp12: P323L S: A222V S: D614G	[97, 150]
GRY	5’ UTR: C241T * Nsp3: F106F Nsp12: P323L S: H69del S: V70del S: Y144del S: N501Y S: D614G N: R203K N: G204R	[89, 146]
O	Variants without mutations characteristic of other clades	[93, 94]

Characteristic mutations for SARS-CoV-2 clades at the amino acid or nucleotide (*) levels

Clade G is characterized in part by the single nucleotide polymorphism (SNP) A23403G within subdomain 2 of the S1 gene, resulting in amino acid mutation D614G [103, 104] (Fig. 2, Table 2). D614G is now detected globally in B.1.1.7, B,1,351, P.1, B.1.617.2 and other variants [97, 104, 105] and increases the infectivity of SARS-CoV-2 by increasing respiratory viral load [106, 107], possibly due to increased S openness [108, 109] or cleavability [110], causing this mutation to become dominant upon emergence [93, 111, 112]. There is also an epidemiological correlation between D614G and anosmia (loss of smell) [109], potentially due to greater viral loads in the olfactory epithelium. Preliminary evidence suggests that D614G increases viral susceptibility to neutralization [113], with uncertain impacts on disease severity [104, 114, 115].

D614G is usually accompanied by three other mutations which represent clade G [104, 116, 117] (Table 3). Of these mutations, P323L in the RNA-dependent RNA polymerase (RdRp), encoded by *Nsp12* (Fig. 2, Table 2), is particularly interesting as CoV RdRp tends to be highly conserved by purifying selection given its critical role in viral genome replication [118, 119] (Table 4). P323 falls outside of the RdRp catalytic site and within a relatively uncharacterized interface domain that may interact with proteins that regulate viral polymerase function [120]. The correlation of this mutation with increased point mutations [121] elsewhere in the genome raises an intriguing hypothesis that P323L diminishes RdRp proofreading ability, leading to increased mutation rates. Moreover, P323L downregulates the association of Nsp12 with the Nsp8 primase subunit (Table 4), reducing polymerase activity and viral replication [122]. Decreased replication could decrease symptomology, leading to reduced COVID-19 detection and greater population-level spread. It is important to characterize the cumulative effect of all mutations, as any reduction in transmission due to P323L could be compensated for by the co-existing D614G mutation. Multiple factors may contribute to the success of clade G and its derivatives via rapid spread with low detection in human populations [104].

**Table 4:** Putative functions of SARS-CoV-2 proteins

Gene	Protein	Putative function	References
Nsp1	Leader protein/host translation inhibitor	Inhibits translation of host mRNAs and promotes expression of viral genes	[320]
Nsp2	Non-structural protein 2	Modulates host cell survival signalling pathways	[321]
Nsp3	Papain-like protease	Proteolytic cleavage of polyprotein to generate Nsps 1–3, and inhibition of host IFN responses	[322, 323]
Nsp4	Non-structural protein 4	Interacts with Nsp3 and host proteins to induce cytoplasmic autophagosomes for viral replication	[324, 325]
Nsp5	Chymotrypsin-like protease	Proteolytic cleavage of polyprotein to generate Nsps 4–16 and mediation of Nsp maturation	[326, 327]
Nsp6	Non-structural protein 6	Interferes with delivery of viral factors to host lysosomes and inhibits IFN-1 responses	[127, 128]
Nsp7	Primase complex	Forms a complex with Nsp8 which interacts with RdRp (Nsp12) to transcribe viral genome	[120]
Nsp8	Primase complex	Forms a complex with Nsp7 which interacts with RdRp (Nsp12) to transcribe viral genome	[120]
Nsp9	ssRNA-binding protein	Binds to viral ssRNA and promotes replication	[328]
Nsp10	Non-structural protein 10	Interacts with 3′–5′ exoribonuclease (Nsp14) and 2′ O-ribose methyltransferase (Nsp16) and promotes methylation of viral mRNA caps	[328]
Nsp11	Non-structural protein 11	Released from cleavage of pp1a and forms N-terminal sequence of Nsp12 in pp1ab frameshift product. No known function	[328]
Nsp12	RNA-dependent RNA polymerase (RdRp)	Replicates and transcribes viral genome	[326]
Nsp13	Helicase	Unwinds dsRNA and dsDNA in viral replication	[326, 329]
Nsp14	3′–5′ exoribonuclease/N7-guanine methyltransferase	Proofreading during RNA replication (exoribonuclease) and viral mRNA capping (methyltransferase). Interacts with Nsp10	[330]
Nsp15	Nidoviral uridylate-specific endoribonuclease	RNA processing and inhibition of host IFN responses	[331]
Nsp16	2′ O-ribose methyltransferase	Activated by Nsp10 for methylation of viral mRNA caps	[332]
S	Spike glycoprotein	Cleaved into S1 and S2 subunits. S1 binds host receptor (ACE2) while S2 mediates viral and host membrane fusion	[333]
ORF3a	Orf3a viroporin	Activates NF-kB and NLRP3 inflammasome to contribute to cytokine storm. Promotes viral release and may induce necrotic cell death	[334–336]
ORF3b	Accessory protein ORF3b	IFN-1 antagonist	[337]
E	Envelope protein	A viroporin involved in viral assembly, budding, and pathogenesis. Forms CoV envelope	[338, 339]
M	Membrane protein	Forms viral membrane and induces N and S localization to the ER-Golgi-Intermediate compartment for virion assembly and budding	[340]
ORF6	Accessory protein ORF6	IFN-1 antagonist	[144]
ORF7a	Accessory protein ORF7a	SARS-CoV ortholog inhibits bone marrow stromal antigen 2 mediated tethering of virions to host plasma membrane	[341]
ORF7b	Accessory protein ORF7b	SARS-CoV ortholog attenuates viral replication	[342]
ORF8	Accessory protein ORF8	Inhibits IFN-1 activity and downregulates MHC-1 expression to evade host immunity	[136, 137, 144]
N	Nucleocapsid	Involved in immune evasion through IFN-1 antagonism, nucleocapsid formation, viral RNA replication, and virion assembly	[144, 145]
ORF9b	Accessory protein ORF9b	Suppresses IFN-1 responses through inhibition of TOM70	[343]
ORF9c	Accessory protein ORF9c	Interferes with IFN signalling, antigen presentation, and complement signalling. Induces IL-6 signalling	[344]
ORF10	Accessory protein ORF10	Interacts with a Cullin 2 RING E3 ligase complex to potentially modulate ubiquitination	[345]

Findings are based on studies with SARS-CoV-2 proteins or SARS-CoV orthologs

Positively selected for residues within SARS-CoV-2 Nsp6 [74, 123–126] are intriguing since Nsp6 is relatively conserved in other coronaviruses [126] (Fig. 2, Table 2)*.* SARS-CoV-2 Nsp6 inhibits IFN-1 responses [127] and may reduce delivery of viral factors to host lysosomes similar to its SARS-CoV ortholog [128] (Table 4). The Nsp6 L37F mutation may impair Nsp6 function [129], decreasing viral replication and causing increased asymptomatic infections [130]. A similar homoplasy occurs in MERS-CoV Nsp6 [74, 131] (Fig. 2), although the outcome of this mutation is unknown. The associated clade V mutation (Table 3) in ORF3a (G251V) reduces viral replication through decreased SARS-CoV-2 intraviral ORF3a-S and ORF3a-membrane protein (M) binding affinity [132]. Nsp6 (L37F) and ORF3a (G251V) mutations were likely selected to decrease pathogenicity and disease severity. A separate positively selected ORF3a mutation (Q57H) [111] characteristic of clade GH variants (Table 3) is speculated to increase ORF3a-S and ORF3a-M binding affinity, promoting virus replication [132]. The ORF3a viroporin is essential for SARS-CoV-2 pathogenesis [133] and limits apoptosis in infected cells relative to its SARS-CoV ortholog [134], potentially contributing to less severe disease outcomes.

Another mutation of interest (L84S) lies within ORF8 [123, 124, 135], a protein implicated in evasion of host immune responses [136, 137] (Table 4). ORF8 was under strong directional selection at the beginning of both SARS-CoV-2 [124] and SARS-CoV outbreaks [138], supporting the theory that it facilitates zoonotic transmission and adaptation in alternate hosts [139, 140]. However, the over-representation of ORF8 deletions in SARS-CoV with no apparent effect on viral survival [138] suggests that ORF8 may be dispensable in humans [139], and L84S mutations may not be significant. While L84S may be important in SARS-CoV-2 virulence and pathogenesis given ORF8’s role in attenuation of host immunity (Table 4), the continued decline of L84S representation among global SARS-CoV-2 sequences [93] suggests otherwise.

Mutations RG203KR within SARS-CoV-2 nucleoprotein (N) have become dominant and characteristic of clade GR [123]. RG203KR alters N protein morphology, resulting in increased intraviral protein binding affinity [132]. N-M interactions are necessary for CoV viral assembly [141, 142], while N-envelope (E) interactions potentially increase production of virus-like particles [143]. Therefore, increased intraviral N protein binding affinities could contribute to increased viral replication. RG203KR may also confer immune evasion properties to SARS-CoV-2 considering the rapid expansion of clade GR and the role of N protein in antagonizing human antiviral immune responses [144, 145] (Table 4). The global prevalence of variant B.1.1.7 has generated clade GRY from clade GR [146].

Clade GV is associated with the European variant 20A.EU1 containing spike NTD mutation A222V [105, 147]. A222 is located within a speculated B lymphocyte epitope [148] that may impact neutralization by human antibodies, consistent with observed SARS-CoV-2 re-infection with a clade GV variant [149]. The rise in prevalence of variant 20A.EU1 and clade GV is most likely associated with the relaxing of travel-associated restrictions across Europe near the end of the summer of 2020 considering the rapid decline in prevalence of global clade GV sequences in 2021 [97, 150].

## Ongoing SARS-CoV-2 evolution and the rise of variants of concern

An aforementioned trend across HCoVs is positively selected residues within RBD [84, 85, 88, 138, 139, 151–154] (Fig. 2, Table 2), which facilitates interactions with host cellular proteins, providing a crucial target for the host immune response [155]. Accordingly, SARS-CoV-2 RBD is rapidly evolving, leading to novel variants [156, 157] (Fig. 2, Table 2). SARS-CoV-2 variants associated with greater transmissibility, altered virulence, or the ability to escape natural infection- and vaccine-mediated immunity or current diagnostic tests are called Variants of Concern (VOC; Table 5).

**Table 5:** SARS-CoV-2 variants of concern (as of July 22, 2021)

Variant	Mutations of interest	Clade	Date of emergence	First detection in human population	Country of likely origin	References
B.1.1.7 (VOC2020 12/01, 501.V1, Alpha)	S: 69-70del S: Y144del S: N501Y S: D614G S: P681H	GRY	September, 2020	December, 2020	United Kingdom	[89, 99, 190]
B.1.351 (501.V2, Beta)	S: K417N S: E484K S: N501Y S: D614G S: A701V	GH	October, 2020	December, 2020	South Africa	[100, 101, 190]
P.1 (501.V3, Gamma)	S: K417T S: E484K S: N501Y S: D614G	GR	July, 2020	January, 2021	Brazil	[101, 190, 194]
B.1.617.2 (Delta)	S: L452R S: T478K S: D614G S: P681R	G	October, 2020	December, 2020	India	[196, 201, 202, 346]

Variant names are based on Rambaut et al*.*’s classification [347]. Other commonly used names are mentioned in brackets. Mutations mentioned here are non-synonymous mutations that are speculated to confer some functional significance. These variants contain other mutations that may also contribute to viral advantages [89, 99–101]. Updated information about SARS-CoV-2 VOCs can be accessed through the GISAID resource (https://www.gisaid.org). Dates of emergence are based on retrospective analyses. S, spike. del, deletion

Early data suggest that RBD mutation N501Y emerged recurrently in multiple regions due to increased transmissibility, and is associated with multiple VOCs [89, 99, 100, 158] (Table 5). SARS-CoV-2 N501 serves as one of six critical S residues required for binding to ACE2 [159] and N501Y increases viral infectivity through greater S-hACE2 binding affinity, likely due to stronger interactions with ACE2 residues Y41 and K353 [160]. Other critical residues within the SARS-CoV-2 RBD (L455, F486, Q493, S494, Y505) [73] should be closely monitored as mutations may increase SARS-CoV-2 transmission in humans and facilitate zooanthroponotic transfer to other species.

Early studies of the highly transmissible B.1.1.7 variant [77, 161] originating in the United Kingdom described 17 co-occurring non-synonymous mutations or deletions [89], which are more than expected since the mutation rate of SARS-CoV-2 is estimated to be around 2.4 × 10^–3^ per site per year [135]. In addition to N501Y, spike 69-70del, Y144del, and P681H mutations are speculated to be of functional significance [78, 162] (Table 5). Spike NTD 69-70del variants have shown significant transmission expansion, with speculated increased resistance to antibody-mediated neutralization [92] likely associated with sequestration of a protruding spike loop [78]. Y144del confers antibody resistance due to loss of a negative surface charge [163, 164]. Spike P681 is located in a known CoV mutational hotspot [83, 101] directly adjacent to the SARS-CoV-2 S1/S2 furin cleavage site (aa 681–684) [89, 165, 166] which promotes virus entry into host cells [167]; mutation in this region may increase cleavability and membrane fusion to enhance infectivity. P681 is also within an antigenic epitope recognized by B and T lymphocytes, implicating host immune response alterations [168]. P681H may therefore represent adaptive evolution to evade host immunity, although confirmatory studies are required. Another speculated B.1.1.7 mutation at ORF8 (Q27stop) causes early protein termination [89]. Truncated ORF8 has been associated with milder symptoms [169], although increased mortality is also associated with the B.1.1.7 variant [79, 170]. Emerging mutations in B.1.1.7 must be monitored and investigated, such as the sub-lineage VOC202102/02 that contains the RBD mutation E484K, which is associated with antibody resistance [171–173].

Another variant containing N501Y is B.1.351, which was first detected in South Africa in December, 2020 [100], but likely originated in October, 2020 [101]. This variant contains eight non-synonymous mutations in S, including three within the RBD (K417N, E484K, N501Y) and three in the NTD that may contribute to increased transmissibility [100, 101]. Both N501Y and E484K are located within the receptor binding motif (RBM) of the RBD. E484 interacts with residue K31 on hACE2 [174], one of two critical hACE2 RBD-interacting residues [159, 175], suggesting that E484K may affect the binding affinity of SARS-CoV-2 with hACE2. However, preliminary studies demonstrate contradictory binding affinity observations [176, 177]; further studies are required. In addition, E484K confers some resistance to antibody-mediated neutralization of SARS-CoV-2 in vitro [91, 154, 178–181], consistent with the observation that E484 is an important recognition site for neutralizing antibodies [181, 182], and raising concerns about E484K being an immune escape mutation appearing in multiple independent SARS-CoV-2 lineages [172, 183–186]. Similarly, spike K417 is within a neutralizing antibody epitope [100]. Preliminary evidence suggests K417N reduces recognition by human antibodies [187]. K417N may impact RBD-hACE2 binding affinity and stabilize E484K, though these effects remain uncertain [91, 177, 187, 188].

Mutations within the RBD (K417T, E484K, N501Y) have also been observed in the P.1 variant (Table 5) that likely originated in Brazil and has since spread to other countries [101, 189–191]. In contrast, the P.2 variant only contains E484K, likely acquired through convergent evolution with P.1 [186, 192]. Little is known about the P.1 variant, but based on emerging data [193], we speculate that the RBD mutations likely affect antibody-mediated neutralization and contribute to increased transmission as observed with B.1.351. Mutations shared between the B.1.1.7, B.1.351, and P.1 variants are speculated to have arisen independently, indicating convergent evolution [194] (Table 5). These variants also share Nsp6 3675-3677del, with unknown functional significance [194, 195].

VOC B.1.617.2 was first identified in India in late 2020 and contains positively selected for mutations within the spike protein, namely, L452R, T478K, and P681R, along with the D614G mutation [196] (Table 5). Mutation of the uncharged and hydrophobic leucine (L) residue into the positively charged and hydrophilic arginine (R) residue at spike position 452 allows for an increased electrostatic interaction with negatively charged ACE2 residues E35, E37, and D38, likely leading to the observed increase in S-hACE2 complex stability, viral infectivity, and virus replication [196, 197]. Furthermore, abolition of the hydrophobic surface patch through the L452R mutation led to reduced antibody-mediated neutralization and cellular immune recognition [196–198]. Spike mutation T478K has also been shown to increase electrostatic interactions in the S-hACE2 complex and may increase binding affinity similar to the S477N mutation [199]. The mutation T478K is within a neutralizing epitope close to the immune evasion mutation E484K/Q that is present in multiple SARS-CoV-2 variants, including the ancestral B.1.617 lineage and current sub-lineages B.1.617.1 and B.1.617.2 [181, 200, 201]. T478K in combination with L452R may contribute to increased resistance to neutralization by monoclonal antibodies, convalescent sera, and vaccinated sera [201, 202]. B.1.617.2 has increased replication efficiency in human airway systems relative to the B.1.1.7 lineage due to enhanced spike cleavability, which is likely augmented by the P681R mutation [201, 203]. P681R is known to increase cell-to-cell fusion in the respiratory tract, potentially increasing transmissibility and pathogenicity in infected individuals [201, 203]. B.1.617.2 may thus represent a VOC with similar resistance to antibody neutralization as B.1.351 and transmissibility beyond B.1.1.7 [200]. Recently discovered B.1.617.2 sequences containing the K417N mutation (AY.1/AY.2 lineages) must be monitored for altered antibody resistance and increased transmissibility [204].

Circulating variants containing an N439K mutation (e.g. B.1.141 and B.1.258) also show some degree of neutralization evasion [91, 198, 205], raising speculations about SARS-CoV-2 variants escaping vaccine-mediated immunity. Emerging data suggest that antibodies elicited by mRNA vaccines (BNT162b2 and mRNA-1273) have 20% and 16.7% reduced neutralization capacity, respectively, against the B.1.1.7 variant [206, 207] and 67% and 84% reduced neutralization capacity, respectively, against the B.1.351 variant [208, 209]. Neutralization capacity of sera from BNT162b2 and mRNA-1273 vaccinated individuals have 87% and 52% reduced neutralization capacity, respectively, against the B.1.617.2 variant [200, 201, 210]. The emergence of B.1.1.7 sub-lineages containing the E484K RBD mutation (e.g. VOC202102/02) pose additional challenges for vaccine-mediated immunity [171, 173, 183]. While complete vaccine failure is unlikely [206, 207, 211–215], immune escape variants may create a need to update current SARS-CoV-2 vaccines. Monitoring the emergence of novel SARS-CoV-2 variants is especially important as vaccine-mediated immunity provides stronger selective pressure for SARS-CoV-2 evolution.

## Other variants of interest

Multiple emerging SARS-CoV-2 lineages are not considered VOCs but are still of interest and may become VOCs in the future. One variant, B.1.525, was first detected in December, 2020, in the United Kingdom and Nigeria and has since spread internationally. B.1.525 contains spike mutations 69-70del, E484K, Q677H, and F888L. Q677P/H has emerged in disparate variants and may affect spike cleavability similar to P681H [158, 216–218]. F888L lies between the fusion peptide and heptad repeat region of the S2 subunit [219] and may impact host cellular entry, similar to the impact of heptad repeat mutations in MERS-CoV [139, 220].

Variant B.1.526 from New York contains spike mutations D253G, D614G, and A701V, along with either E484K or S477N, creating two major B.1.526 sub-lineages. NTD mutation D253G reduces antibody-mediated neutralization [163]. A701V, shared by variant B.1.351 [100], is in the S2 subunit adjacent to the furin cleavage site [219] and may impact SARS-CoV-2 cleavability and infectivity. S477N, also found in variant 20A.EU2, increases binding to hACE2 [221, 222] and reduces antibody-mediated neutralization [178, 223], likely due to its position within a neutralizing epitope [224]. D614G and E484K are shared with multiple other variants (Table 5) and likely play a role in B.1.526 expansion.

P681H found in variant B.1.1.207 from Nigeria [162] may enhance infectivity and modulate host immunity as speculated for B.1.1.7. Similar effects are expected for P681R in variant A.23.1 that emerged in Uganda [183, 225]. The UK A.23.1 sub-lineage VUI-202102/01 also contains immune escape mutation E484K [171, 183]. Preliminary data show increased ACE2 binding affinity and reduced antibody-mediated neutralization for the P.3 variant from Brazil, which contains the spike mutations E484K, N501Y, and P681H [164]. Data also suggest increased ACE2 binding affinity and reduced neutralization profile for the B.1.620 variant from Central Africa, which contains spike mutations E484K, S477N, D614G, and P681H [226]. Other notable variants include N440K variants from India [227] that have increased transmissibility, and the R.1 variant from Japan which contains potential immune escape mutations W152L and E484K [228].

B.1.427/B.1.429 are two emerging lineages that originated in California in May 2020 [229], however, circulating B.1.427/B.1.429 variants are now being replaced by more transmissible variants, such as B.1.1.7 and B.1.617.2 [97, 230]. B.1.427/B.1.429 contains multiple positively selected for mutations within the S protein, such as S13I, W152C, and L452R, all of which contribute to some degree of resistance to antibody-mediated neutralization [229]. L452R has convergently evolved in the B.1.617 lineage and contributed to enhanced SARS-CoV-2 infectivity [196–198] (Table 5). Spike mutation L452Q was detected in the recently emerged C.37 lineage from Peru and is expected to have similar impacts on virus infectivity as the L452R mutation [231]. C.37 also shares Nsp6 3675-3677del with B.1.1.7, B.1.351. and P.1 variants [231], and contains the spike RBD mutation F490S that has been associated with reduced antibody-mediated neutralization [91, 178]. These variants need to be monitored for transmission expansion and convergent evolution.

## Multiple factors will determine the evolutionary trajectory of SARS-CoV-2 and the COVID-19 pandemic

The future of SARS-CoV-2 and COVID-19 remains uncertain. Many virological, immunological, and social factors will influence the epidemiological trajectory of this virus. One particularly intriguing question that remains unanswered is whether SARS-CoV-2 will become endemic in the human population, like HCoVs NL63, OC43, HKU1, and 229E [232–234].

Currently, endemic HCoVs cause seasonal outbreaks [235], with increased circulation observed in the winter in temperate regions [232]. Cold temperatures are favourable for enveloped viruses [236], as lower temperatures enhance lipid ordering of the viral envelope, allowing the virus to remain protected outside the host for longer periods of time [237, 238]. Low temperatures also enhance aerosol transmission of respiratory viruses by allowing virions to remain suspended in the air for a longer duration [239]. Furthermore, cold and dry environments can have immunosuppressive effects on a potential host, further increasing the chances of infection [240–242]. Evidence suggests decreased transmission of SARS-CoV-2 in warmer climates [243–246], likely due to degeneration of viral structural stability with increasing temperatures [247]. Decreased transmission of SARS-CoV-2 was not observed during the summer of 2020 [11, 248] likely because of the sheer number of cases and an immunologically naïve population. For seasonality to have an observable impact on SARS-CoV-2 transmission, the basic reproduction number (R_0_) must drop from its current estimate of around 2.5 to less than 1 [249]. In theory, SARS-CoV-2 R_0_ should drop substantially when population herd immunity is reached through natural infection and vaccination, allowing for meteorological factors to influence viral transmission, leading to seasonal fluctuations. Other intervention mechanisms such as effective social distancing, quarantine, and contact-tracing will contribute towards reducing the R_0_ for SARS-CoV-2 [250, 251].

Multiple studies have demonstrated short-lasting immunity to endemic HCoVs, with waning of protective immunity and re-infections common within 80 days [85] to one year [252–255]. There is no observable association between endemic HCoV re-infection and infection severity [254]. Waning of humoral immunity within a year [256–260] and re-infection of immunocompetent patients [149] have been demonstrated for SARS-CoV-2, suggesting the possibility of annual outbreaks [233, 261]. A weaker initial immune response and sharper decline of antibody levels have been reported in individuals with asymptomatic SARS-CoV-2 infections [257, 258]. Thus, multiple exposures to SARS-CoV-2 may be required to develop sufficient immunity to prevent future re-infections, which may also be influenced by adaptive evolution of SARS-CoV-2 in the human population (Table 5). The duration of protection through vaccination and natural exposures is being closely monitored, along with antigenic evolution of SARS-CoV-2 that may lead to immune escape. Indeed, the evolutionary trajectories of endemic HCoVs suggest that SARS-CoV-2 will evolve to co-exist with the human population. However, with roll-out of the first ever HCoV vaccines, predicting the evolutionary trajectory of SARS-CoV-2 remains challenging.

An important factor that may influence ongoing SARS-CoV-2 transmission is the potential for cross-protection by humoral and cellular immune responses induced by related endemic HCoVs. There is evidence of cross-protection within the same genera of HCoVs [233, 262, 263], but not between genera [264]. Thus, immunity against *beta-CoVs* HCoV-OC43 and HCoV-HKU1 may provide some protection against COVID-19 [265–268], while immunity against *alpha-CoVs* HCoV-229E and HCoV-NL63 will likely provide little to no protection. Antibody-dependent enhancement has not been observed for SARS-CoV-2 [269, 270], ruling out the possibility of increased disease severity by cross-reactive antibodies generated against endemic HCoVs. The high frequency of CoV recombination during co-infections raises the additional concern that SARS-CoV-2 recombination with seasonal HCoVs could generate novel CoVs [131, 271, 272]. The role of HCoV co-infection has not been reported or extensively studied and will be especially important for immunocompromised and elderly individuals.

## Conclusions

SARS-CoV-2 continues to evolve and adapt to the human population as highlighted by the emergence of novel variants. Mutations within the spike protein of SARS-CoV-2 variants confer increased transmissibility and some degree of resistance to antibody-mediated neutralization. However, recurrent attenuating mutations, such as P323L, L37F, G251V, and Q27stop have also been identified and are speculated to reduce disease severity. The appearance of attenuating mutations suggests that SARS-CoV-2 is evolving to become less pathogenic in humans. The current SARS-CoV-2 pandemic is driven by asymptomatic, pre-symptomatic, or otherwise unrecognized cases [273–275]. Reduced pathogenicity of SARS-CoV-2 combined with mounting population-level immunity will likely cause a reduction of severe cases of COVID-19, leading to an apparent abatement of the pandemic, followed by endemic circulation of low pathogenic SARS-CoV-2 variants. A similar evolutionary trajectory may have led to the establishment of current low-pathogenic endemic HCoVs [276].

Monitoring future emerging variants of SARS-CoV-2 is critical to determine control measures for the COVID-19 pandemic. Mutations speculated to reduce immune recognition, such as within the spike protein (S13I, 69-70del, W152L, A222V, K417N, N439K, S477N, T478K, E484K/Q, F490S, P681H/R) and nucleoprotein (RG203KR) should be studied for reduced sensitivity to natural or vaccine-induced immunity. Other factors, such as zoonotic and zooanthroponotic transmission of SARS-CoV-2, cross-protection through immunity against endemic HCoVs, and the possible creation of novel animal reservoirs through zooanthroponosis should continue to be investigated as they may have significant implications on the evolutionary trajectory of SARS-CoV-2 and the COVID-19 pandemic.

## Data Availability

Not applicable.

## Abbreviations

CoV: Coronavirus
*dN*: Rate of nonsynonymous mutations
*dS*: Rate of synonymous mutations
E: Envelope protein
hACE2: Human ACE2
HCoV: Human coronavirus
HE: Hemagglutinin esterase
M: Membrane protein
MERS-CoV: Middle East respiratory syndrome coronavirus
N: Nucleocapsid protein
S: Spike glycoprotein
NTD: N-terminal domain
PEDV: Porcine epidemic diarrhea virus
RdRp: RNA-dependent RNA polymerase
RBD: Receptor binding domain
RBM: Receptor binding motif
R_0_: Basic reproduction number
SADS-CoV: Swine acute diarrhea syndrome coronavirus
SARS-CoV: Severe acute respiratory syndrome coronavirus
SARS-CoV-2: Severe acute respiratory syndrome coronavirus 2
SARSr-CoV: Severe acute respiratory syndrome related coronavirus
SNP: Single nucleotide polymorphism
VOC: Variant of concern
